# Hybrid Chains: A Collaboration of Ubiquitin and Ubiquitin-Like Modifiers Introducing Cross-Functionality to the Ubiquitin Code

**DOI:** 10.3389/fchem.2019.00931

**Published:** 2020-01-22

**Authors:** David A. Pérez Berrocal, Katharina F. Witting, Huib Ovaa, Monique P. C. Mulder

**Affiliations:** Department of Cell and Chemical Biology, Chemical Immunology, Leiden University Medical Center, Oncode Institute, Leiden, Netherlands

**Keywords:** ubiquitin-like modifiers, hybrid chains, SUMO and ubiquitin signaling, NEDD8, ISG15, proteotoxic conditions, stress conditions, toolbox

## Abstract

The Ubiquitin CODE constitutes a unique post-translational modification language relying on the covalent attachment of Ubiquitin (Ub) to substrates, with Ub serving as the minimum entity to generate a message that is translated into different cellular pathways. The creation of this message is brought about by the dedicated action of writers, erasers, and readers of the Ubiquitin CODE. This CODE is greatly expanded through the generation of polyUb chains of different architectures on substrates thus regulating their fate. Through additional post-translational modification by Ub-like proteins (UbL), hybrid Ub/UbL chains, which either alter the originally encrypted message or encode a completely new one, are formed. Hybrid Ub/UbL chains are generated under both stress or physiological conditions and seem to confer improved specificity and affinity toward their cognate receptors. In such a manner, their formation must play a specific, yet still undefined role in cellular signaling and thus understanding the UbCODE message is crucial. Here, we discuss the evidence for the existence of hybrid Ub/UbL chains in addition to the current understanding of its biology. The modification of Ub by another UbL complicates the deciphering of the spatial and temporal order of events warranting the development of a hybrid chain toolbox. We discuss this unmet need and expand upon the creation of tailored tools adapted from our previously established toolkit for the Ubiquitin Proteasome System to specifically target these hybrid Ub/UbL chains.

## Introduction

**Figure 1:**
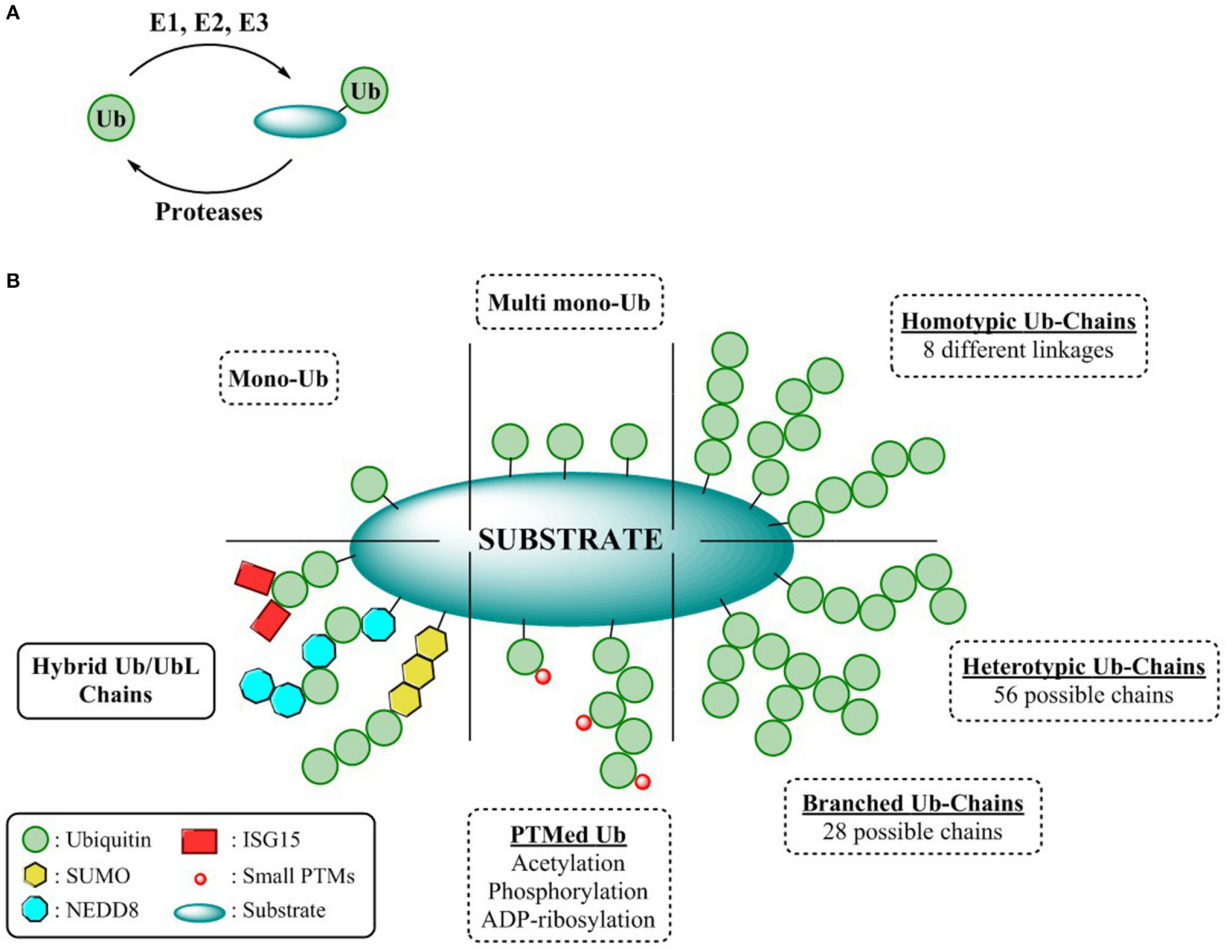
Complexity of the ubiquitin CODE **(A)** General overview ubiquitination process. **(B)** Ubiquitin chain types.

Ubiquitin (Ub) is a 76 amino acid, highly conserved protein among eukaryotes post-translationally modifying proteins thereby dictating almost every fundamental cellular process. Malfunction of its action drives diverse pathologies such as cancer and neurological disorders like Parkinson's, Alzheimer's, and Huntington's disease (McNaught et al., 2001; Du and Mei, 2013; Ciechanover and Kwon, 2015). It exerts its action through the covalent attachment of its C-terminus to the target substrates by an orchestrated enzymatic cascade composed by three different enzyme families named E1, E2, E3 (writers of the code) (Figure 1A). This conjugation process, commonly referred to as ubiquitination, is initiated once the E1 activating enzyme catalyzes adenylation of the C-terminus of Ub at the expense of ATP thereby forming a high-energy E1-Ub thioester. Afterwards, the activated Ub is transferred by trans-thioesterification to the cysteine of the E2 conjugating enzyme which allows E3 ligase mediated Ub conjugation the substrate lysine residue through a stable peptide bond. Ub transfer to the substrate can be carried out by three different mechanisms depending on the nature of participating E3 ligase [RING, HECT, and RING-in-between-RING (RBR)] (Zheng and Shabek, 2017). Activated Ub can be transferred onto the catalytic cysteine of the HECT E3s via a transthioesterification reaction followed by conjugation to the lysine residue of the substrate. Alternatively, transfer of the E2-Ub thioester to the substrate lysine is accomplished by the contribution of a scaffolding RING E3 enzyme accommodating both the E2-Ub complex and the substrate. RBR E3s catalyze Ub conjugation by a concerted RING/HECT hybrid mechanism in which the RING1 domain recruits the E2-Ub complex, followed by thioester transfer of Ub to a cysteine in the RING2 domain (Spratt et al., 2014).

Different ubiquitination patterns can be observed depending on the constitution of the lysine residues of the substrate, giving rise to mono-ubiquitination or multi mono-ubiquitination, respectively. Additionally, this enzymatic process can be repeated by utilizing the ε-amine functionality of any of the seven internal lysine residues or the N-terminal amine of Ub. Thus, self-conjugation of Ub to any of these residues permits the formation of eight different homogenous polymeric Ub chains (M1, K6/11/27/29/33/48/63). Due to the different disposition adopted by each of these Ub linkages, a wide variety of cellular signaling (Akutsu et al., 2016) events can be modulated all exerting different biological outcomes. For instance, Lys-48 and Lys-63 linked poly-Ub, the best characterized polymeric chains are mainly involved in proteasome mediated protein degradation and cell signaling respectively, whereas the cellular responses of the remaining linkages, known as atypical chains, remains undefined (Kulathu and Komander, 2012). Furthermore, complexity can be augmented through permutation of linkages, either through modification of different internal lysines (branched chains) or by repetition of different linkages within the chain (heterologous/mixed chains) thereby endowing the UbCODE with an extraordinary versatility and specificity (Nakasone et al., 2013; Stolz and Dikic, 2018; Haakonsen and Rape, 2019) (Figure 1B).

To counterbalance ubiquitination and further sculpting the physiological effects or rescuing proteins destined for proteasomal degradation, dedicated proteases known as deubiquitinases (DUBs) not only remove mono-Ub from their substrates, but also alter Ub chain topology (editors of the code) (Leznicki and Kulathu, 2017). Alternatively, modulating the formation and processing of Ub chains can be achieved through their interaction with Ubiquitin Binding Domains (UBDs). These UBDs are endowed with a specific affinity toward Ub or Ub chains permitting the modulation of both chain elongation as well as governing the interaction of the Ub chains with the substrates (Dikic et al., 2009).

While Ubiquitin represents the minimum entity to express a code, the Ubiquitin CODE as coined by Komander and Rape, it is a highly complex, yet still elusive signaling system reliant on the interplay of its “writers,” “editors,” and “readers” (Komander and Rape, 2012). Nonetheless, intricacy arises when Ub, is further “PTMylated” by the classical modifications such as acetylation (Ohtake et al., 2015), phosphorylation (Herhaus and Dikic, 2015), or ribosylation (Vivelo et al., 2019), or even by some Ubiquitin-like proteins (UbL). Structurally and biochemically similar to Ub, UbLs are covalently attached to the lysine residues of their substrates through the sequential action of dedicated activating, conjugating, and ligating enzymes. Conjugation of UbLs to Ub and vice versa, results in hybrid chains, expanding the utility of the Ubiquitin CODE to enable an extensive crosstalk among the different UbL pathways and the UPS (Schmidt and Dikic, 2006; Schimmel et al., 2008; Geoffroy and Hay, 2009; Hjerpe et al., 2012a) (Figure 1B). However, the assembly, topology, architecture, as well as the encoded information of these Ub/UbLs hybrid chains remains cryptic warranting the development of suitable reagents to decipher this intricate CODE.

Given the breadth of this review, we will focus on evidence supporting the existence of these Hybrid Chains with ubiquitin-like modifiers mainly composed of Ub and the UbL proteins NEDD8, SUMO, and ISG15 as well as the future potential for this emerging field. Additionally, we will touch upon the crosstalk between the Ubiquitin and the Ubiquitin-like enzyme cascades that cooperate to form hybrid Ub/UbL chains.

## Ubiquitin-Like Proteins and Hybrid Chain Formatio

### Small Ubiquitin-Related Modifier (SUMO)

SUMOylation, which is involved in a large plethora of fundamental cellular processes, is catalyzed through the interplay of specific enzymes and counteracted by the action of SUMO specific isopeptidases (Pichler et al., 2017). The SUMO family is composed by three different members known as SUMO-1, -2, and -3, which, subsequent to the exposure of their C-terminal di-glycine signature, are conjugated onto specific lysines embedded within a SUMO consensus motif (ψ-Lys-X-Glu, with ψ encoding a hydrophobic residue of their substrates) (Geiss-Friedlander and Melchior, 2007). While the most predominant isoforms SUMO-2 and SUMO-3 are virtually identical and mainly form K11-linked polymeric chains (Matic et al., 2008; Hendriks et al., 2014), SUMO-1 bears only a 50% sequence similarity and does not form polymeric chains give the absence of the necessary conserved lysine residue within the consensus motif (Saitoh and Hinchey, 2000). However, it has been shown that SUMO-1 can be linked to the end of a poly-SUMO-2/-3 chain, effectively terminating chain growth (Matic et al., 2008). Formation of SUMO-2/-3 chains is elicited upon cellular stressors such as heat shock (Saitoh and Hinchey, 2000) and their recognition is mediated by SUMO interactive motifs (SIMs)- specific regions interacting with SUMO and SUMO polymers (Song et al., 2004).

#### Hybrid SUMO-Ub Chains

In addition to modification with SUMO itself, several proteomic studies have identified that Ubiquitination at various lysines in SUMO-1–3 can occur (Danielsen et al., 2011; Wagner et al., 2012; Hendriks et al., 2014; Hendriks and Vertegaal, 2016). Interestingly, while SUMO-1 cannot be SUMOylated, it is Ubiquitinated at six lysine residues most likely inducing a different response than Ubiquitinated SUMO-2/3 (Hendriks and Vertegaal, 2016). Thus, given the sheer number of Ubiquitination sites in SUMO a plethora of hybrid chains combinations are possible.

**Figure 2:**
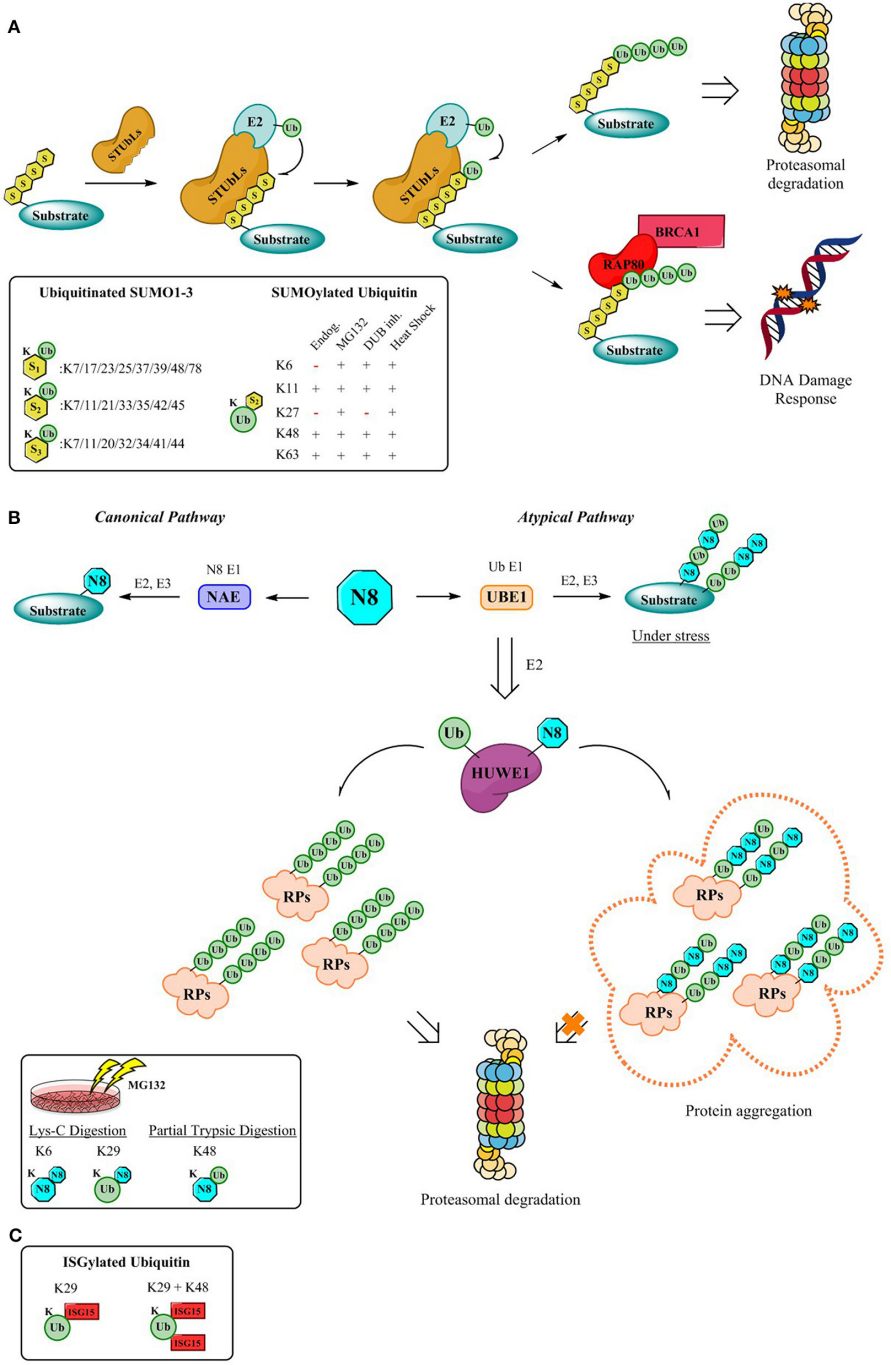
Mechanism for the formation of hybrid chains. **(A)** StubLs containing tandem of SIMS recognize polySUMO2/3 chains and poly-ubiquitinate in a linkage specific manner the PolySUMO chains targeting them for enhanced proteasomal degradation (Aillet et al., 2012) or initiates signaling for DDR events through the RAP80/BRCA1 complex (Guzzo et al., 2012). The insert highlights ubiquitinitated SUMO1-3 (Hendriks and Vertegaal, 2016) and SUMOylated Ubiquitin identified by proteomics so far (Hendriks et al., 2014). **(B)** Canonical and atypical pathways (Leidecker et al., 2012) and dual activity of HUWE1 under stress conditions which lead to formation of hybrid chains which protect the UPS by via the formation of aggregates that are no longer processed by the proteasome (Maghames et al., 2018). The insert displays identified Ub-Nedd8 linkages (Leidecker et al., 2012). **(C)** Although the mechanism for the formation of ISG15 hybrid chains is still outstanding, data supporting the existence of Ub-ISG15 is available. First, K29 gets ISGylated, followed by K48 as the second ISG15 site (Fan et al., 2015).

Intriguingly, proteomics revealed not only the vast number of modification possibilities on the different SUMO isoforms, but also allowed to identify whether the modification occurs on SUMO or on the Ubiquitin lysines (Hendriks et al., 2014, 2017), further increasing the complexity (Figure 2A). The hybrid chains predominantly occur upon specific stressors (Hendriks et al., 2014) (Figure 2A) and despite the advances in detection and elucidation of the branched architecture of SUMO-Ub hybrid chains, comprehending their cellular function is still in its infancy. Discerning their physiological roles is of utmost importance since the architecture of hybrid SUMO-Ub chains expands the potential for distinct signaling events by SUMO and Ub.

#### SUMO-Ub Chain Signaling

Hybrid chains can be recognized by a variety of receptors containing tandem SUMO-interacting motifs (SIMs) and UBDs. Moreover, to counterbalance or alter the effect of SUMOylation, subsequent ubiquitination of poly-SUMOylated proteins, catalyzed by SUMO-targeted Ubiquitin ligases (STUbls) can ensue. Upon recognition of the poly-SUMO signal through virtue of their SIMs, STUbls install a specific Ub-linkage onto the lysine of the SUMO-modification (Sriramachandran and Dohmen, 2014). Although, SUMO-Ub chains were primarily identified on proteins impending proteasomal degradation (Lallemand-Breitenbach et al., 2008; Tatham et al., 2008; Erker et al., 2013; McIntosh et al., 2018), roles in maintaining genome stability (Guzzo et al., 2012; Nie and Boddy, 2016) have been assigned more recently through the K63 poly-ubiquitination of poly-SUMO chains (Figure 2A). Here RNF4, a STUbl, mediates poly-ubiquitination of SUMOylated proteins, thereby evoking the recruitment of RAP80 and its subsequent interaction with BRCA1 complex, to promote genomic stability (Guzzo et al., 2012). Another example involves Arkadia which ubiquitinates SUMOylated xeroderma pigmentosum C (XPC), a pivotal player in nucleotide excision repair, driving XPC to UV-damaged DNA sites (Poulsen et al., 2013).

DUBs such as USP11 can trim or reverse ubiquitination on hybrid SUMO-Ub chains to modulate the associated cellular responses (Hendriks et al., 2015). Thus, the amalgamation of ubiquitination and SUMOylation resembles an efficacious strategy to confer both specificity and increased affinity to the target proteins (Aillet et al., 2012; Guzzo et al., 2012).

### Neural Precursor Cell Expressed, Developmentally Downregulated 8 (NEDD8)

Akin to SUMOylation, Neddylation is accomplished by its own specific enzymes and is counterbalanced by a few dedicated proteases (Enchev et al., 2015). Given the similarities between Ubiquitin and Nedd8, it is unsurprising that both have the propensity to form hybrid chains. However, the formation of Ubiquitin-Nedd8 hybrid chains occurs predominantly in response to proteotoxic stress, perhaps as a mechanism to dampen cellular signaling in this context or to protect the UPS from proteotoxicity (Maghames et al., 2018; Santonico, 2019). Neddylation and the Nedd8 enzyme cascade have been demonstrated to be crucial to the development of neurological disorders (Dil Kuazi et al., 2003; Mori et al., 2005; Chen et al., 2012; Lu et al., 2013). Thus, given the protective role of Ubiquitin-Nedd8 hybrid chains against proteotoxic stress, these complex posttranslational modifications may play a pivotal role in the pathogenesis and progression of neurodegenerative diseases (Ross and Poirier, 2004; Gestwicki and Garza, 2012; Dantuma and Bott, 2014; Valastyan and Lindquist, 2014; Sweeney et al., 2017).

In an attempt to elucidate the architecture of the hybrid chains several hybrid linkages were determined by SILAC-based proteomics upon proteasomal inhibition (Leidecker et al., 2012) (Figure 2B). Neddylation occurs via the interplay of enzymes relying on its own specificity and is referred to as the “Canonical” pathway. However, under stress conditions such as proteasome inhibition, oxidative stress, or heat shock Neddylation is mediated “atypically” by the Ubiquitin activating enzyme UBE1 instead (Hjerpe et al., 2012a,b; Leidecker et al., 2012). This tight synchronization of the Ubiquitin and Nedd8 systems to fine-tune the cellular response during proteotoxic stress has been observed not only for UBE1, but also for also for the E3 ligase HUWE1, a crucial component of the Protein Quality Control (PQC) pathway (Xirodimas et al., 2008; Sung et al., 2016a,b), which targets ribosomal proteins (RPs) and protects the UPS from stress-induced toxicity by ribosomal protein aggregation (Maghames et al., 2018) (Figure 2B). Importantly, during the persistence of stress, the unconjugated Ub pool is rapidly depleted triggering Neddylation through the Ubiquitin pathway and targeting several substrates typically ubiquitinated (Leidecker et al., 2012) (Figure 2B). Akin to the sophisticated regulatory system provided by DUBs, research underscores that Nedd8-Ub hybrid chains seem to be modulated in a similar fashion by DUBs subsequent to cellular stress (Leidecker et al., 2012; Singh et al., 2012, 2014).

### Interferon (IFN)-Stimulated Gene 15 (ISG15)

Firstly identified upon IFN treatment on Ehrlich ascites tumor cells (Farrell et al., 1979), ISG15 had initially not been identified as an Ubiquitin-like protein, until cross-reactivity toward Ub antibodies suggested the existence of UbL proteins (Haas et al., 1987). Unlike all other UbLs, ISG15 is composed of two Ub like domains tethered by a “hinge” polypeptide sequence. Analogous to Ub, ISG15 can be conjugated onto the target substrates through the orchestrated interplay of its E1, E2, and E3 enzymes through its exposed C-terminal glycine (Perng and Lenschow, 2018). Given its increased activation upon interferon stimulation, conjugation of ISG15 to protein substrates plays a crucial role in the antiviral response and thereby constituting a key contributor to innate immunity (Harty et al., 2009; Durfee et al., 2010; Perng and Lenschow, 2018).

In contrast to Ub, SUMO and NEDD8 (Jones et al., 2008), ISG15 has not been reported to generate polymeric chains and does not seem to have specific ISG15-interacting motifs. Although some studies have suggested an antagonistic relationship of Ub and ISG15 in certain contexts such as during tumorigenesis (Liu et al., 2003; Desai et al., 2006; Kim et al., 2006; Malakhova and Zhang, 2008; Wood et al., 2011), evidence of a crosstalk between ISG15 and Ub conjugation pathways still remains perplexing. Unexpectedly, a proteomic study revealed that ISG15 was conjugated to Ub (Giannakopoulos et al., 2005), and further investigation by Zhang et al. corroborated the formation of hybrid ISG15-Ub chains (Fan et al., 2015) (Figure 2C).

Little is known about the biological function of these hybrid Ub-ISG15 chains, but it has been established that they do not act as proteasomal degradation signals. Thus, ISG15 could potentially function as a chain termination moiety to rescue ubiquitylated proteins from degradation. However, given the fact that ISG15 is predominantly conjugated to Ub via K29, a plausible role of these hybrid chains could be modulation of K29-Ub mediated biological signaling (Kulathu and Komander, 2012). Moreover, this type of hybrid chains could trigger new signaling pathways exerting different biological outcomes, but the assignment of their biological role is a daunting task since no ISG15 interactive motifs have been identified and readers containing both UIM and ISG15 interacting motifs cannot be predicted.

## Perspectives

Considering the impact of ubiquitination on regulating a vast array of fundamental biological processes, with dysregulation of the dedicated enzymes giving rise to pathologies such as cancer and neurodegenerative diseases, understanding its function merited the development and innovation of respective tools. Advances in synthetic strategies for generating ubiquitin, constituted a qualitative leap forward in the development of a plethora of ubiquitin assay reagents and numerous activity-based probes (ABPs) enabling study of enzymes involved in the complex system of ubiquitination (El Oualid et al., 2010; Ekkebus et al., 2013; Hameed et al., 2017).

The modification of Ub by another UbL complicates the deciphering of the spatial and temporal order of events, as well as the underlying biological role of this modification, underscoring the urgent need for new next generation ABPs and assay reagents. The lack of a robust methodology to chemically access some UbL proteins has hampered the study on the biological role that hybrid chains display as well as the identification of their readers, writers, erasers, and interpreters. Generating such complex hybrid chains is a challenging feat as the E2/E3 enzymes generating these linkages *in vitro* remain unknown. So far, only (semi)-synthetic strategies for obtaining ubiquitinated Rub1, the yeast NEDD8 homolog (Singh et al., 2014) and SUMO-2-K63diUb hybrid chains (Bondalapati et al., 2017) have been reported. Only in the last decade, efforts to devise synthetic strategies for UbL proteins such as Nedd8 (Mulder et al., 2014), SUMO (Dobrota et al., 2012; Wucherpfennig et al., 2014; Mulder et al., 2018) and Ufm1 (Ogunkoya et al., 2012; Witting et al., 2018) have been undertaken. More recently, ISG15 synthesis has been accomplished as a modular synthesis of both domains and its subsequent ligation (Xin et al., 2019). These developments in the chemical synthesis of UbL proteins in combination with the advancements made in polyUb probes (Mulder et al., 2014; Flierman et al., 2016; Paudel et al., 2019) open a new avenue to UbL and hybrid Ub/UbL reagents allowing research on their respective enzymatic cascades, but also enabling in depth studies on their crosstalk with ubiquitin.

Mass spectrometry (MS) has become an invaluable tool in the quest for understanding cell signaling and in particular to study the UPS (Heap et al., 2017). This type of proteomics relies on the isolation and enrichment of the target proteins through affinity-based approaches (Mattern et al., 2019) such as affimers, antibodies targeting the di-Glycine signature, anti/mini/nanobodies, endogenous tags, biotin, and molecular entities based in the repetition of UBDs and SIMs capturing poly-Ub and SUMO chains, respectively (TUBES and SUBES) (Hjerpe et al., 2009; Da Silva-Ferrada et al., 2013) with a high affinity. However, many of these approaches cannot be undertaken in the study toward hybrid Ub-UbL biology since they are not endowed with specific affinity toward these linkages or due to the shared homology under Ub and UbL proteins as exemplified by the shared GG remnant after enzymatic digestion. To overcome these pitfalls, an UbiSite antibody approach (Akimov et al., 2018) which relies on LysC digestion has recently been described to allow differentiation among Ub and UbL proteins. The translation of the existing affinity technologies toward hybrid chains and UbL proteins would facilitate the understanding of the crosstalk among the different Ub-UbL proteins. For example, an elegant combination of SIMs and UBDs, a mixed TUBE/SUBE approach, could potentially enrich for substrates endowed with hybrid chains generated by STUbLs. Unsurprisingly due to the high similarity of Nedd8 and Ub, all known binding domains with affinity for Nedd8 display cross-reactivity with Ub. Recently, the first specific binding domain for Nedd8 was reported (Castagnoli et al., 2019) and thus a similar approach as the TUBES/SUBES could potentially be designed, “NEBES.”

Furthermore, a proteomic approach called Ubi-clipping (Swatek et al., 2019) has shown the great percentage (10–20%) of which branched chains are present in polymeric forms of Ub. This method relies on an engineered version of an ISG15-specificenzyme that partially removes Ub from substrates and leave the characteristic diglycine signature on Ub while simultaneously allowing the identification of different branched architectures. The translation of such technology into the hybrid chains field would shed light on the different architectures that such chains exhibit. In addition to this innovation, the generation of specific antibodies toward the linkage of hybrid chains, in a similar fashion as the first Ub branched K11/K48 antibody (Yau et al., 2017) could be a feasible approach toward the generation a Hybrid Chain Tool Box.

Despite the recent advances made in developing innovative reagents on the Ubiquitin-field, there are still many conundrums to be resolved regarding the writers, editors and readers of this part of the Ub CODE. The origin of the identified Ub-SUMO linkages in which Ub is SUMOylated is still unclear, the possibility of a parallel mechanism such as the STUbL in which SUMO ligases target polyUb-chains and SUMOylate (UbTSLs) them might explain their existence. The enzymes catalyzing the formation of Ub-ISG15 hybrid chains are still unknown and efforts to identify them should be undertaken. Moreover, the formation of these hybrid chains confers an extra layer of complexity to the CODE that could be translated into terms of specificity and increased affinity that the “readers” display for them. Such readers must be endowed with “hybrid” recognition domains which could be screened by bioinformatic analysis as exemplified in the discovery of RAP80 (Nie and Boddy, 2016). It has been shown that hybrid chains are processed by the proteasome more efficiently compared with poly-Ub or poly-SUMO chains. This pronounced affinity could be derived from the improved recognition of either a proteasome subunit or of a shuttle protein containing the aforementioned “hybrid” recognition domains. For the Ub-Nedd8 and Ub-ISG15 hybrid chains, the field is less explored and hybrid chain recognition domains still need to be identified.

The fact that Ub and UbL proteins can generate this array of chains, conferring new architectures and topology to the chains and thereby triggering different signaling events, increases complexity of the already intricate Ubiquitin CODE. The current knowledge regarding hybrid-chain formation is based upon chain formation between Ub and UbL proteins. However, a recent report revealed that a small fraction of NEDD8 becomes modified by K0-SUMO (Hendriks et al., 2017). Although SUMOylation of NEDD8 is likely to be a very rare event, it does extend the knowledge regarding hybrid chain cross-talk and opens a new perspective to the intrinsic code (Hendriks et al., 2014). The creation of tailored tools specific toward these hybrid chains by adapting the methodology already applied for the study of the Ubiquitin Proteasome System will augment our knowledge about hybrid chains.

## Author Contributions

DP, KW, and MM wrote sections of the manuscript. All authors contributed to manuscript revision, read, and approved the submitted version.

### Conflict of Interest

HO is shareholder of the reagent company UbiQ Bio BV. The remaining authors declare that the research was conducted in the absence of any commercial or financial relationships that could be construed as a potential conflict of interest.

## References

[B1] Aillet F. Lopitz-Otsoa F. Egaña I. Hjerpe R. Fraser P. Hay R. T.. (2012). Heterologous SUMO-2/3-ubiquitin chains optimize IκBα degradation and NF-κB activity. PLoS ONE 7:e51672. 10.1371/journal.pone.0051672 23284737 PMC3527444

[B2] Akimov V. Barrio-Hernandez I. Hansen S. V. F. Hallenborg P. Pedersen A.-K. Bekker-Jensen D. B.. (2018). UbiSite approach for comprehensive mapping of lysine and N-terminal ubiquitination sites. Nat. Struct. Mol. Biol. 25, 631–640. 10.1038/s41594-018-0084-y 29967540

[B3] Akutsu M. Dikic I. Bremm A. (2016). Ubiquitin chain diversity at a glance. J. Cell Sci. 129, 875–880. 10.1242/jcs.183954 26906419

[B4] Bondalapati S. Eid E. Mali S. M. Wolberger C. Brik A. (2017). Total chemical synthesis of SUMO-2-Lys63-linked diubiquitin hybrid chains assisted by removable solubilizing tags. Chem. Sci. 8, 4027–4034. 10.1039/C7SC00488E 28580118 PMC5434752

[B5] Castagnoli L. Mandaliti W. Nepravishta R. Valentini E. Mattioni A. Procopio R.. (2019). Selectivity of the CUBAN domain in the recognition of ubiquitin and NEDD8. FEBS J. 286, 653–677. 10.1111/febs.14752 30659753

[B6] Chen Y. Neve R. L. Liu H. (2012). Neddylation dysfunction in Alzheimer's disease. J. Cell. Mol. Med. 16, 2583–2591. 10.1111/j.1582-4934.2012.01604.x 22805479 PMC3484225

[B7] Ciechanover A. Kwon Y. T. (2015). Degradation of misfolded proteins in neurodegenerative diseases: therapeutic targets and strategies. Exp. Mol. Med. 47:e147. 10.1038/emm.2014.117 25766616 PMC4351408

[B8] Da Silva-Ferrada E. Xolalpa W. Lang V. Aillet F. Martin-Ruiz I. de la Cruz-Herrera C. F.. (2013). Analysis of SUMOylated proteins using SUMO-traps. Sci. Rep. 3:1690. 10.1038/srep01690 23604351 PMC3631770

[B9] Danielsen J. M. Sylvestersen K. B. Bekker-Jensen S. Szklarczyk D. Poulsen J. W. Horn H.. (2011). Mass spectrometric analysis of lysine ubiquitylation reveals promiscuity at site level. Mol. Cell. Proteomics 10:M110.003590. 10.1074/mcp.M110.003590 21139048 PMC3047152

[B10] Dantuma N. P. Bott L. C. (2014). The ubiquitin-proteasome system in neurodegenerative diseases: precipitating factor, yet part of the solution. Front. Mol. Neurosci. 7:70. 10.3389/fnmol.2014.00070 25132814 PMC4117186

[B11] Desai S. D. Haas A. L. Wood L. M. Tsai Y. C. Pestka S. Rubin E. H.. (2006). Elevated expression of ISG15 in tumor cells interferes with the ubiquitin/26S proteasome pathway. Cancer Res. 66, 921–928. 10.1158/0008-5472.CAN-05-1123 16424026

[B12] Dikic I. Wakatsuki S. Walters K. J. (2009). Ubiquitin-binding domains — from structures to functions. Nat. Rev. Mol. Cell Biol. 10:659. 10.1038/nrm2767 19773779 PMC7359374

[B13] Dil Kuazi A. Kito K. Abe Y. Shin R.-W. Kamitani T. Ueda N. (2003). NEDD8 protein is involved in ubiquitinated inclusion bodies. J. Pathol. 199, 259–266. 10.1002/path.1283 12533840

[B14] Dobrota C. Fasci D. Hadade N. D. Roiban G. D. Pop C. Meier V. M.. (2012). Glycine fluoromethylketones as SENP-specific activity based probes. Chembiochem 13, 80–84. 10.1002/cbic.201100645 22134988

[B15] Du W. Mei Q.-B. (2013). Ubiquitin-proteasome system, a new anti-tumor target. Acta Pharmacol. Sin. 34, 187–188. 10.1038/aps.2012.192 23381108 PMC4011612

[B16] Durfee L. A. Lyon N. Seo K. Huibregtse J. M. (2010). The ISG15 conjugation system broadly targets newly synthesized proteins: implications for the antiviral function of ISG15. Mol. Cell 38, 722–732. 10.1016/j.molcel.2010.05.002 20542004 PMC2887317

[B17] Ekkebus R. van Kasteren S. I. Kulathu Y. Scholten A. Berlin I. Geurink P. P.. (2013). On terminal alkynes that can react with active-site cysteine nucleophiles in proteases. J. Am. Chem. Soc. 135, 2867–2870. 10.1021/ja309802n 23387960 PMC3585465

[B18] El Oualid F. Merkx R. Ekkebus R. Hameed D. S. Smit J. J. de Jong A.. (2010). Chemical synthesis of ubiquitin, ubiquitin-based probes, and diubiquitin. Angew. Chem. 49, 10149–10153. 10.1002/anie.201005995 21117055 PMC3021723

[B19] Enchev R. I. Schulman B. A. Peter M. (2015). Protein neddylation: beyond cullin-RING ligases. Nat. Rev. Mol. Cell Biol. 16, 30–44. 10.1038/nrm3919 25531226 PMC5131867

[B20] Erker Y. Neyret-Kahn H. Seeler J. S. Dejean A. Atfi A. Levy L. (2013). Arkadia, a novel SUMO-targeted ubiquitin ligase involved in PML degradation. Mol. Cell. Biol. 33, 2163–2177. 10.1128/MCB.01019-12 23530056 PMC3648077

[B21] Fan J.-B. Arimoto K.-L. Motamedchaboki K. Yan M. Wolf D. A. Zhang D.-E. (2015). Identification and characterization of a novel ISG15-ubiquitin mixed chain and its role in regulating protein homeostasis. Sci. Rep. 5:12704. 10.1038/srep12704 26226047 PMC4520236

[B22] Farrell P. J. Broeze R. J. Lengyel P. (1979). Accumulation of an mRNA and protein in interferon-treated Ehrlich ascites tumour cells. Nature 279, 523–525. 10.1038/279523a0 571963

[B23] Flierman D. van Noort G. J. V. Ekkebus R. Geurink P. P. Mevissen T. E. T. Hospenthal M. K.. (2016). Non-hydrolyzable diubiquitin probes reveal linkage-specific reactivity of deubiquitylating enzymes mediated by S2 pockets. Cell Chem. Biol. 23, 472–482. 10.1016/j.chembiol.2016.03.009 27066941 PMC4850247

[B24] Geiss-Friedlander R. Melchior F. (2007). Concepts in sumoylation: a decade on. Nat. Rev. Mol. Cell Biol. 8, 947–956. 10.1038/nrm2293 18000527

[B25] Geoffroy M. C. Hay R. T. (2009). An additional role for SUMO in ubiquitin-mediated proteolysis. Nat. Rev. Mol. Cell Biol. 10, 564–568. 10.1038/nrm2707 19474794

[B26] Gestwicki J. E. Garza D. (2012). Protein quality control in neurodegenerative disease. Prog. Mol. Biol. Transl. Sci. 107, 327–353. 10.1016/B978-0-12-385883-2.00003-5 22482455

[B27] Giannakopoulos N. V. Luo J. K. Papov V. Zou W. Lenschow D. J. Jacobs B. S.. (2005). Proteomic identification of proteins conjugated to ISG15 in mouse and human cells. Biochem. Biophys. Res. Commun. 336, 496–506. 10.1016/j.bbrc.2005.08.132 16139798

[B28] Guzzo C. M. Berndsen C. E. Zhu J. Gupta V. Datta A. Greenberg R. A.. (2012). RNF4-dependent hybrid SUMO-ubiquitin chains are signals for RAP80 and thereby mediate the recruitment of BRCA1 to sites of DNA damage. Sci. Signal. 5:ra88. 10.1126/scisignal.2003485 23211528 PMC4131685

[B29] Haakonsen D. L. Rape M. (2019). Branching out: improved signaling by heterotypic ubiquitin chains. Trends Cell Biol. 29, 704–716. 10.1016/j.tcb.2019.06.003 31300189

[B30] Haas A. L. Ahrens P. Bright P. M. Ankel H. (1987). Interferon induces a 15-kilodalton protein exhibiting marked homology to ubiquitin. J. Biol. Chem. 262, 11315–11323. 2440890

[B31] Hameed D. S. Sapmaz A. Ovaa H. (2017). How chemical synthesis of ubiquitin conjugates helps to understand ubiquitin signal transduction. Bioconjug. Chem. 28, 805–815. 10.1021/acs.bioconjchem.6b00140 27077728

[B32] Harty R. N. Pitha P. M. Okumura A. (2009). Antiviral activity of innate immune protein ISG15. J. Innate Immun. 1, 397–404. 10.1159/000226245 19680460 PMC2725329

[B33] Heap R. E. Gant M. S. Lamoliatte F. Peltier J. Trost M. (2017). Mass spectrometry techniques for studying the ubiquitin system. Biochem. Soc. Trans. 45, 1137–1148. 10.1042/BST20170091 28939693

[B34] Hendriks I. A. D'Souza R. C. Yang B. Verlaan-de Vries M. Mann M. Vertegaal A. C. (2014). Uncovering global SUMOylation signaling networks in a site-specific manner. Nat. Struct. Mol. Biol. 21, 927–936. 10.1038/nsmb.2890 25218447 PMC4259010

[B35] Hendriks I. A. Lyon D. Young C. Jensen L. J. Vertegaal A. C. Nielsen M. L. (2017). Site-specific mapping of the human SUMO proteome reveals co-modification with phosphorylation. Nat. Struct. Mol. Biol. 24, 325–336. 10.1038/nsmb.3366 28112733

[B36] Hendriks I. A. Schimmel J. Eifler K. Olsen J. V. Vertegaal A. C. (2015). Ubiquitin-specific protease 11 (USP11). Deubiquitinates hybrid Small Ubiquitin-like Modifier (SUMO)-ubiquitin chains to counteract RING Finger Protein 4 (RNF4). J. Biol. Chem. 290, 15526–15537. 10.1074/jbc.M114.618132 25969536 PMC4477612

[B37] Hendriks I. A. Vertegaal A. C. (2016). A comprehensive compilation of SUMO proteomics. Nat. Rev. Mol. Cell Biol. 17, 581–595. 10.1038/nrm.2016.81 27435506

[B38] Herhaus L. Dikic I. (2015). Expanding the ubiquitin code through post-translational modification. EMBO Rep. 16, 1071–1083. 10.15252/embr.201540891 26268526 PMC4576978

[B39] Hjerpe R. Aillet F. Lopitz-Otsoa F. Lang V. England P. Rodriguez M. S. (2009). Efficient protection and isolation of ubiquitylated proteins using tandem ubiquitin-binding entities. EMBO Rep. 10, 1250–1258. 10.1038/embor.2009.192 19798103 PMC2775171

[B40] Hjerpe R. Thomas Y. Chen J. Zemla A. Curran S. Shpiro N.. (2012a). Changes in the ratio of free NEDD8 to ubiquitin triggers NEDDylation by ubiquitin enzymes. Biochem. J. 441, 927–936. 10.1042/BJ20111671 22004789 PMC3280039

[B41] Hjerpe R. Thomas Y. Kurz T. (2012b). NEDD8 overexpression results in neddylation of ubiquitin substrates by the ubiquitin pathway. J. Mol. Biol. 421, 27–29. 10.1016/j.jmb.2012.05.013 22608973

[B42] Jones J. Wu K. Yang Y. Guerrero C. Nillegoda N. Pan Z. Q.. (2008). A targeted proteomic analysis of the ubiquitin-like modifier nedd8 and associated proteins. J. Proteome Res. 7, 1274–1287. 10.1021/pr700749v 18247557 PMC2676899

[B43] Kim K. I. Yan M. Malakhova O. Luo J. K. Shen M. F. Zou W. (2006). Ube1L and protein ISGylation are not essential for alpha/beta interferon signaling. Mol. Cell. Biol. 26, 472–479. 10.1128/MCB.26.2.472-479.2006 16382139 PMC1346917

[B44] Komander D. Rape M. (2012). The ubiquitin code. Annu. Rev. Biochem. 81, 203–229. 10.1146/annurev-biochem-060310-170328 22524316

[B45] Kulathu Y. Komander D. (2012). Atypical ubiquitylation - the unexplored world of polyubiquitin beyond Lys48 and Lys63 linkages. Nat. Rev. Mol. Cell Biol. 13, 508–523. 10.1038/nrm3394 22820888

[B46] Lallemand-Breitenbach V. Jeanne M. Benhenda S. Nasr R. Lei M. Peres L.. (2008). Arsenic degrades PML or PML-RARalpha through a SUMO-triggered RNF4/ubiquitin-mediated pathway. Nat. Cell Biol. 10, 547–555. 10.1038/ncb1717 18408733

[B47] Leidecker O. Matic I. Mahata B. Pion E. Xirodimas D. P. (2012). The ubiquitin E1 enzyme Ube1 mediates NEDD8 activation under diverse stress conditions. Cell Cycle 11, 1142–1150. 10.4161/cc.11.6.19559 22370482

[B48] Leznicki P. Kulathu Y. (2017). Mechanisms of regulation and diversification of deubiquitylating enzyme function. J. Cell Sci. 130, 1997–2006. 10.1242/jcs.201855 28476940

[B49] Liu M. Li X. L. Hassel B. A. (2003). Proteasomes modulate conjugation to the ubiquitin-like protein, ISG15. J. Biol. Chem. 278, 1594–1602. 10.1074/jbc.M208123200 12426315

[B50] Lu B. Al-Ramahi I. Valencia A. Wang Q. Berenshteyn F. Yang H. (2013). Identification of NUB1 as a suppressor of mutant Huntingtin toxicity via enhanced protein clearance. Nat. Neurosci. 16:562 10.1038/nn.3367 23525043

[B51] Maghames C. M. Lobato-Gil S. Perrin A. Trauchessec H. Rodriguez M. S. Urbach S.. (2018). NEDDylation promotes nuclear protein aggregation and protects the Ubiquitin Proteasome System upon proteotoxic stress. Nat. Commun. 9:4376. 10.1038/s41467-018-06365-0 30349034 PMC6197266

[B52] Malakhova O. A. Zhang D. E. (2008). ISG15 inhibits Nedd4 ubiquitin E3 activity and enhances the innate antiviral response. J. Biol. Chem. 283, 8783–8787. 10.1074/jbc.C800030200 18287095 PMC2276364

[B53] Matic I. van Hagen M. Schimmel J. Macek B. Ogg S. C. Tatham M. H.. (2008). *In vivo* Identification of human small ubiquitin-like modifier polymerization sites by high accuracy mass spectrometry and an *in vitro* to *in vivo* strategy. Moll Cell Proteomics 7, 132–144. 10.1074/mcp.M700173-MCP200 17938407 PMC3840926

[B54] Mattern M. Sutherland J. Kadimisetty K. Barrio R. Rodriguez M. S. (2019). Using ubiquitin binders to decipher the ubiquitin code. Trends Biochem. Sci. 44, 599–615. 10.1016/j.tibs.2019.01.011 30819414

[B55] McIntosh D. J. Walters T. S. Arinze I. J. Davis J. (2018). Arkadia (RING finger protein 111). Mediates sumoylation-dependent stabilization of Nrf2 through K48-linked ubiquitination. Cell. Physiol. Biochem. 46, 418–430. 10.1159/000488475 29597191 PMC6595223

[B56] McNaught K. S. P. Olanow C. W. Halliwell B. Isacson O. Jenner P. (2001). Failure of the ubiquitin–proteasome system in Parkinson's disease. Nat. Rev. Neurosci. 2, 589–594. 10.1038/35086067 11484002

[B57] Mori F. Nishie M. Piao Y.-S. Kito K. Kamitani T. Takahashi H.. (2005). Accumulation of NEDD8 in neuronal and glial inclusions of neurodegenerative disorders. Neuropathol. Appl. Neurobiol. 31, 53–61. 10.1111/j.1365-2990.2004.00603.x 15634231

[B58] Mulder M. P. C. El Oualid F. ter Beek J. Ovaa H. (2014). A native chemical ligation handle that enables the synthesis of advanced activity-based probes: diubiquitin as a case study. Chembiochem 15, 946–949. 10.1002/cbic.201402012 24623714 PMC4159580

[B59] Mulder M. P. C. Merkx R. Witting K. F. Hameed D. S. El Atmioui D. Lelieveld L.. (2018). Total chemical synthesis of SUMO and SUMO-based probes for profiling the activity of SUMO-specific proteases. Angew. Chem. 57, 8958–8962. 10.1002/anie.201803483 29771001 PMC6055820

[B60] Nakasone M. A. Livnat-Levanon N. Glickman M. H. Cohen R. E. Fushman D. (2013). Mixed-linkage ubiquitin chains send mixed messages. Structure 21, 727–740. 10.1016/j.str.2013.02.019 23562397 PMC3654000

[B61] Nie M. Boddy M. N. (2016). Cooperativity of the SUMO and ubiquitin pathways in genome stability. Biomolecules 6:14. 10.3390/biom6010014 26927199 PMC4808808

[B62] Ogunkoya A. O. Pattabiraman V. R. Bode J. W. (2012). Sequential alpha-ketoacid-hydroxylamine (KAHA). ligations: synthesis of C-terminal variants of the modifier protein UFM1. Angew. Chem. 51, 9693–9697. 10.1002/anie.201204144 22915333

[B63] Ohtake F. Saeki Y. Sakamoto K. Ohtake K. Nishikawa H. Tsuchiya H.. (2015). Ubiquitin acetylation inhibits polyubiquitin chain elongation. EMBO Rep. 16, 192–201. 10.15252/embr.201439152 25527407 PMC4328746

[B64] Paudel P. Zhang Q. Leung C. Greenberg H. C. Guo Y. Chern Y.-H.. (2019). Crystal structure and activity-based labeling reveal the mechanisms for linkage-specific substrate recognition by deubiquitinase USP9X. Proc. Natl. Acad. Sci. U.S.A. 116, 7288–7297. 10.1073/pnas.1815027116 30914461 PMC6462090

[B65] Perng Y. C. Lenschow D. J. (2018). ISG15 in antiviral immunity and beyond. Nat. Rev. Microbiol. 16, 423–439. 10.1038/s41579-018-0020-5 29769653 PMC7097117

[B66] Pichler A. Fatouros C. Lee H. Eisenhardt N. (2017). SUMO conjugation - a mechanistic view. Biomol. Concepts 8, 13–36. 10.1515/bmc-2016-0030 28284030

[B67] Poulsen S. L. Hansen R. K. Wagner S. A. van Cuijk L. van Belle G. J. Streicher W.. (2013). RNF111/Arkadia is a SUMO-targeted ubiquitin ligase that facilitates the DNA damage response. J. Cell Biol. 201, 797–807. 10.1083/jcb.201212075 23751493 PMC3678163

[B68] Ross C. A. Poirier M. A. (2004). Protein aggregation and neurodegenerative disease. Nat. Med. 10, S10–S17. 10.1038/nm1066 15272267

[B69] Saitoh H. Hinchey J. (2000). Functional heterogeneity of small ubiquitin-related protein modifiers SUMO-1 versus SUMO-2/3. J. Biol. Chem. 275, 6252–6258. 10.1074/jbc.275.9.6252 10692421

[B70] Santonico E. (2019). New insights into the mechanisms underlying NEDD8 structural and functional specificities, in Ubiquitin Proteasome System Current Insights into Mechanism Cellular Regulation and Disease (IntechOpen). Available online at: https://www.intechopen.com/books/ubiquitin-proteasome-system-current-insights-into-mechanism-cellular-regulation-and-disease

[B71] Schimmel J. Larsen K. M. Matic I. van Hagen M. Cox J. Mann M.. (2008). The ubiquitin-proteasome system is a key component of the SUMO-2/3 cycle. Mol. Cell. Proteomics 7, 2107–2122. 10.1074/mcp.M800025-MCP200 18565875

[B72] Schmidt M. H. H. Dikic I. (2006). Ubiquitin and NEDD8: brothers in arms. Sci. STKE 2006:pe50. 10.1126/stke.3622006pe50 17119158

[B73] Singh R. K. Sundar A. Fushman D. (2014). Nonenzymatic rubylation and ubiquitination of proteins for structural and functional studies. Angew. Chem. 53, 6120–6125. 10.1002/anie.201402642 24764216 PMC4128492

[B74] Singh R. K. Zerath S. Kleifeld O. Scheffner M. Glickman M. H. Fushman D. (2012). Recognition and cleavage of related to ubiquitin 1 (Rub1). and Rub1-ubiquitin chains by components of the ubiquitin-proteasome system. Mol. Cell. Proteomics 11, 1595–1611. 10.1074/mcp.M112.022467 23105008 PMC3518131

[B75] Song J. Durrin L. K. Wilkinson T. A. Krontiris T. G. Chen Y. (2004). Identification of a SUMO-binding motif that recognizes SUMO-modified proteins. Proc. Natl. Acad. Sci. U.S.A. 101, 14373–14378. 10.1073/pnas.0403498101 15388847 PMC521952

[B76] Spratt D. E. Walden H. Shaw G. S. (2014). RBR E3 ubiquitin ligases: new structures, new insights, new questions. Biochem. J. 458, 421–437. 10.1042/BJ20140006 24576094 PMC3940038

[B77] Sriramachandran A. M. Dohmen R. J. (2014). SUMO-targeted ubiquitin ligases. Biochim. Biophys. Acta 1843, 75–85. 10.1016/j.bbamcr.2013.08.022 24018209

[B78] Stolz A. Dikic I. (2018). Heterotypic ubiquitin chains: seeing is believing. Trends Cell Biol. 28, 1–3. 10.1016/j.tcb.2017.11.005 29191367

[B79] Sung M. K. Porras-Yakushi T. R. Reitsma J. M. Huber F. M. Sweredoski M. J. Hoelz A.. (2016a). A conserved quality-control pathway that mediates degradation of unassembled ribosomal proteins. eLife 5:e19105. 10.7554/eLife.19105.026 27552055 PMC5026473

[B80] Sung M. K. Reitsma J. M. Sweredoski M. J. Hess S. Deshaies R. J. (2016b). Ribosomal proteins produced in excess are degraded by the ubiquitin-proteasome system. Mol. Biol. Cell 27, 2642–2652. 10.1091/mbc.e16-05-0290 27385339 PMC5007085

[B81] Swatek K. N. Usher J. L. Kueck A. F. Gladkova C. Mevissen T. E. T. Pruneda J. N.. (2019). Insights into ubiquitin chain architecture using Ub-clipping. Nature 572, 533–537. 10.1038/s41586-019-1482-y 31413367 PMC6823057

[B82] Sweeney P. Park H. Baumann M. Dunlop J. Frydman J. Kopito R.. (2017). Protein misfolding in neurodegenerative diseases: implications and strategies. Transl. Neurodegener. 6:6. 10.1186/s40035-017-0077-5 28293421 PMC5348787

[B83] Tatham M. H. Geoffroy M. C. Shen L. Plechanovova A. Hattersley N. Jaffray E. G.. (2008). RNF4 is a poly-SUMO-specific E3 ubiquitin ligase required for arsenic-induced PML degradation. Nat. Cell Biol. 10, 538–546. 10.1038/ncb1716 18408734

[B84] Valastyan J. S. Lindquist S. (2014). Mechanisms of protein-folding diseases at a glance. Dis. Models Mech. 7, 9–14. 10.1242/dmm.013474 24396149 PMC3882043

[B85] Vivelo C. A. Ayyappan V. Leung A. K. L. (2019). Poly(ADP-ribose)-dependent ubiquitination and its clinical implications. Biochem. Pharmacol. 167, 3–12. 10.1016/j.bcp.2019.05.006 31077644 PMC6702056

[B86] Wagner S. A. Beli P. Weinert B. T. Schölz C. Kelstrup C. D. Young C.. (2012). Proteomic analyses reveal divergent ubiquitylation site patterns in murine tissues. Mol. Cell. Proteomics 11, 1578–1585. 10.1074/mcp.M112.017905 22790023 PMC3518112

[B87] Witting K. F. van der Heden van Noort G. J. Kofoed C. Talavera Ormeno C. El Atmioui D. Mulder M. P. C.. (2018). Generation of the UFM1 toolkit for profiling UFM1-specific proteases and ligases. Angew. Chem. 57, 14164–14168. 10.1002/anie.201809232 30188611 PMC6220884

[B88] Wood L. M. Sankar S. Reed R. E. Haas A. L. Liu L. F. McKinnon P.. (2011). A novel role for ATM in regulating proteasome-mediated protein degradation through suppression of the ISG15 conjugation pathway. PLoS ONE 6:e16422. 10.1371/journal.pone.0016422 21298066 PMC3027683

[B89] Wucherpfennig T. G. Pattabiraman V. R. Limberg F. R. Ruiz-Rodriguez J. Bode J. W. (2014). Traceless preparation of C-terminal alpha-ketoacids for chemical protein synthesis by alpha-ketoacid-hydroxylamine ligation: synthesis of SUMO2/3. Angew. Chem. 53, 12248–12252. 10.1002/anie.201407014 25244549

[B90] Xin B.-T. Gan J. Fernandez D. J. Knobeloch K.-P. Geurink P. P. Ovaa H. (2019). Total chemical synthesis of murine ISG15 and an activity-based probe with physiological binding properties. Org. Biomol. Chem. 17, 10148–10152. 10.1039/C9OB02127B 31710063

[B91] Xirodimas D. P. Sundqvist A. Nakamura A. Shen L. Botting C. Hay R. T. (2008). Ribosomal proteins are targets for the NEDD8 pathway. EMBO Rep. 9, 280–286. 10.1038/embor.2008.10 18274552 PMC2267383

[B92] Yau R. G. Doerner K. Castellanos E. R. Haakonsen D. L. Werner A. Wang N.. (2017). Assembly and function of heterotypic ubiquitin chains in cell-cycle and protein quality control. Cell 171, 918–933.e20. 10.1016/j.cell.2017.09.040 29033132 PMC5669814

[B93] Zheng N. Shabek N. (2017). Ubiquitin ligases: structure, function, and regulation. Annu. Rev. Biochem. 86, 129–157. 10.1146/annurev-biochem-060815-014922 28375744

